# Biomimetic dexamethasone-loaded nanoparticles attenuate sepsis-induced acute lung injury

**DOI:** 10.3389/fphar.2026.1857409

**Published:** 2026-07-29

**Authors:** LiuGuang Song, ZheChu Xuan, ChunNa Jin, LiangLiang Jia, Nan Zheng, YuMeng Liu, ZhengMing Wu, Jian Shen, Huijun Liang

**Affiliations:** 1 Department of Cardiology, The Second Affiliated Hospital, Zhejiang University School of Medicine, Hangzhou, China; 2 State Key Laboratory of Transvascular Implantation Devices, Hangzhou, China; 3 Heart Regeneration and Repair Key Laboratory of Zhejiang Province, Hangzhou, China; 4 School of Medicine and Nursing, Huzhou University, Huzhou, China; 5 Zhejiang Provincial People’s Hospital, Affiliated People’s Hospital, Hangzhou Medical College, Hangzhou, China; 6 The Third People’s Hospital of Deqing, Deqing Hospital of Hangzhou Normal University, Deqing, China; 7 Laboratory Department, The Second Hospital of Jiaxing, Jiaxing, China

**Keywords:** acute lung injury, biomimetic nanocarriers, dexamethasone, inflammation, PLGA nanoparticles, sepsis

## Abstract

**Background:**

Sepsis-induced acute lung injury (ALI) is a severe complication characterized by uncontrolled inflammation and high mortality, yet effective targeted therapies remain limited. Notably, acute myocardial infarction (MI) frequently coexists with or exacerbates ALI through shared inflammatory pathways. Dexamethasone (Dex) exerts potent anti-inflammatory effects but its clinical application is limited by non-specific biodistribution and dose-dependent off-target toxicities.

**Methods:**

We developed a biomimetic nanoplatform Dex@mPLGA by coating dexamethasone-loaded poly (lactic-co-glycolic acid) (PLGA) nanoparticles with RAW264.7 macrophage membranes. The physicochemical properties of Dex@mPLGA were systematically characterized. Cellular uptake, cytotoxicity, anti-inflammatory, antioxidant, anti-apoptotic, and immunomodulatory effects were evaluated in LPS-stimulated RAW264.7 cells. *In vivo* biodistribution, therapeutic efficacy, and biosafety were further evaluated in an LPS-induced ALI mouse model.

**Results:**

Dex@mPLGA exhibited a core–shell structure with an average particle size of 147.3 nm, negative surface charge, 3.2% drug loading, 74.6% encapsulation efficiency, good colloidal stability, and sustained Dex release. SDS-PAGE analysis supported the retention of macrophage membrane protein components in the final formulation. Dex@mPLGA was internalized by RAW264.7 macrophages in a time-dependent manner and showed minimal cytotoxicity at the tested concentrations. In LPS-stimulated macrophages, Dex@mPLGA alleviated inflammatory and oxidative stress-related responses relative to the LPS model, including improved cell viability, reduced ROS and MDA levels, decreased pro-inflammatory cytokine and NO production, increased IL-10 secretion, reduced apoptosis, restored proliferative activity, and modulation of macrophage polarization. Direct comparison with uncoated Dex@PLGA showed that the additional *in vitro* benefit of macrophage membrane coating was endpoint-dependent rather than universal. Statistically significant coating-associated improvements were mainly observed in cell viability, IL-10 secretion, EdU-positive proliferative recovery, and M2 polarization, whereas several inflammatory, oxidative stress-related, apoptotic, and M1-polarization readouts were comparable between Dex@PLGA and Dex@mPLGA. *In vivo*, Dex@mPLGA showed improved pulmonary accumulation in inflamed lung tissues, improved survival outcomes, reduced systemic pro-inflammatory cytokine levels, and alleviated lung histopathological injury. Short-term biosafety evaluation showed no obvious organ toxicity or abnormal serum biochemical changes under the tested conditions.

**Conclusion:**

Dex@mPLGA represents an innovative biomimetic Dex delivery platform with therapeutic potential for sepsis-induced ALI. Its efficacy is likely associated with PLGA-mediated sustained Dex delivery, macrophage-associated uptake, selected membrane-related immunoregulatory benefits, and improved pulmonary accumulation *in vivo*. However, the incremental *in vitro* advantage of Dex@mPLGA over Dex@PLGA was modest and endpoint-dependent, and the membrane coating should be interpreted as providing selected auxiliary benefits rather than uniformly enhancing all biological outcomes. Further studies are needed to validate membrane-specific targeting and long-term safety in more physiologically relevant models.

## Introduction

Sepsis is a life-threatening condition caused by a dysregulated host response to infection, resulting in systemic inflammation and multiple organ dysfunction syndrome (MODS) (Cao et al., 2023; Liu et al., 2024). It affects millions of people worldwide and is associated with high mortality, particularly in patients with septic shock (La Via et al., 2024). Acute lung injury (ALI) is a common and severe complication of sepsis, characterized by diffuse pulmonary edema, inflammatory cell infiltration, alveolar barrier disruption, and hyaline membrane formation (Li et al., 2022; Qiao et al., 2024; Sun et al., 2023). Despite advances in anti-infective and supportive therapies, effective interventions that directly modulate the excessive inflammatory cascade in sepsis-induced ALI remain limited (Cheng et al., 2025). Therefore, a deeper understanding of the inflammatory mechanisms underlying sepsis-induced ALI and the development of improved anti-inflammatory delivery strategies are important for enhancing therapeutic outcomes.

Excessive macrophage activation is recognized as a key driver of the inflammatory cascade during the development of sepsis-induced ALI (Wang and Wang, 2023; Li et al., 2025). Upon sensing pathogen-associated molecular patterns (PAMPs), such as lipopolysaccharide (LPS), and damage-associated molecular patterns (DAMPs) released from injured cells, alveolar macrophages and recruited monocyte-derived macrophages are rapidly activated and tend to polarize toward a pro-inflammatory M1-like phenotype (Furnari et al., 2025). Activated macrophages secrete a broad range of inflammatory mediators, including tumor necrosis factor-α (TNF-α), interleukin-6 (IL-6), interleukin-1β (IL-1β), and monocyte chemoattractant protein-1 (MCP-1) (Abdollahi et al., 2023). These mediators amplify the cytokine storm, promote inflammatory-cell recruitment, disrupt the alveolar epithelial–endothelial barrier, and contribute to impaired gas exchange (Zhang J. et al., 2024). Moreover, inflammatory signals can sustain macrophage activation through autocrine and paracrine pathways, creating a positive feedback loop that aggravates pulmonary inflammation and tissue injury (Rodríguez-Morales and Franklin, 2023). Excessive inflammation is also closely associated with oxidative stress, characterized by the overproduction of reactive oxygen species (ROS) and lipid peroxidation products, which further exacerbates cellular and tissue damage in ALI (Tan et al., 2022; Zheng et al., 2026).

Dexamethasone (Dex), a well-established synthetic glucocorticoid, exerts potent anti-inflammatory activity by activating the glucocorticoid receptor (GR), which subsequently suppresses key inflammatory transcription pathways, including nuclear factor-κB (NF-κB) signaling (Zhang et al., 2023). This mechanism reduces pro-inflammatory cytokine expression and mitigates excessive inflammatory responses. Dex has been widely used in severe inflammatory diseases and acute respiratory distress syndrome, a severe manifestation of ALI (Gorman et al., 2022). However, its clinical application is limited by nonspecific biodistribution and dose-dependent adverse effects. Systemic administration may lead to drug exposure in non-target tissues, contributing to metabolic disturbances such as enhanced gluconeogenesis, dysregulated lipid metabolism, and glucose intolerance (Madam et al., 2022). In addition, prolonged or high-dose glucocorticoid treatment may induce immunosuppression and increase the risk of secondary infections. Therefore, improving Dex delivery to pulmonary inflammatory lesions while reducing nonspecific systemic exposure is an important strategy for enhancing its therapeutic index in sepsis-induced ALI.

Poly (lactic-co-glycolic acid) (PLGA) is an FDA-approved biodegradable polymer with favorable biocompatibility, tunable degradation kinetics, and controllable drug-release properties, making it widely used in nanomedicine-based drug delivery systems (Kırımlıoğlu, 2023). By optimizing physicochemical properties such as particle size, surface charge, and hydrophobicity, PLGA nanoparticles can be internalized by cells of the mononuclear phagocyte system and can serve as sustained drug-release carriers (Woodworth et al., 2026). Previous studies have shown that negatively charged PLGA nanoparticles with a hydrodynamic diameter of approximately 150 nm can be efficiently internalized by pro-inflammatory macrophages and suppress the expression of inflammatory mediators such as IL-6 and TNF-α *in vitro* (Puricelli et al., 2023). However, conventional PLGA nanoparticles still face limitations, including nonspecific clearance by the mononuclear phagocyte system, predominant liver and spleen accumulation, and limited delivery efficiency to pulmonary inflammatory sites (Wu et al., 2022). These limitations may restrict their therapeutic efficacy in sepsis-induced ALI.

To address these challenges, biomimetic nanocarriers have attracted increasing attention in recent years (Al-Hetty et al., 2023). Coating nanoparticles with natural cell membranes can provide biological surface features that are difficult to fully reproduce using synthetic materials, such as improved biocompatibility, immune camouflage, and potential inflammatory-site interaction (Zhang Z. et al., 2024). Macrophage-derived membranes contain a variety of membrane proteins and adhesion-related molecules that may contribute to interactions with inflammatory microenvironments (Wang et al., 2025). Such biomimetic coating strategies may improve nanoparticle accumulation at inflammatory sites and enhance macrophage-associated uptake, although the specificity and mechanisms of membrane-mediated targeting require careful validation using appropriate controls (Savchenko et al., 2023).

Based on this rationale, we developed a biomimetic system composed of dexamethasone-loaded PLGA nanoparticles coated with RAW264.7 macrophage membranes, referred to as Dex@mPLGA, for the treatment of sepsis-induced ALI. This system integrates the sustained-release capacity of a PLGA core with the biological surface properties of macrophage membrane coating. The PLGA core enables Dex encapsulation and controlled release, whereas the macrophage membrane coating may contribute to improved interaction with macrophage-associated inflammatory microenvironments and enhanced pulmonary accumulation. We hypothesized that Dex@mPLGA could improve Dex delivery in sepsis-induced ALI, thereby attenuating inflammatory cytokine production, reducing oxidative stress, limiting apoptosis, and modulating macrophage polarization.

In this study, we systematically evaluated the physicochemical properties of Dex@mPLGA, including morphology, particle size, zeta potential, drug loading, colloidal stability, membrane protein retention, and *in vitro* Dex release. We further examined its macrophage uptake behavior and assessed its anti-inflammatory, antioxidant, anti-apoptotic, and immunomodulatory effects in an LPS-stimulated RAW264.7 macrophage inflammation model. In addition, we evaluated its biodistribution, pulmonary accumulation, therapeutic efficacy, histopathological improvement, and preliminary biosafety in an LPS-induced sepsis-associated ALI mouse model. This work presents Dex@mPLGA as a promising biomimetic Dex delivery platform for sepsis-induced ALI, while recognizing that membrane-specific targeting and long-term safety require further validation.

## Materials and methods

### Chemicals and biological reagents

Poly (lactic-co-glycolic) acid (PLGA, 50:50, Mw: 50 kDa) and dexamethasone (Dex, purity ≥ 98%) were purchased from Shanghai Yuanye Bio-Technology Co., Ltd. (Shanghai, China). LPS and FITC were purchased from Sigma-Aldrich (St. Louis, MO, USA). The Cell Counting Kit-8 (CCK-8), the EdU cell proliferation assay kit, reactive oxygen species (ROS), malondialdehyde (MDA), and nitric oxide (NO) assay kits were obtained from Beyotime Biotechnology (Shanghai, China). The Annexin V-FITC/PI apoptosis detection kit was purchased from Yeasen (Shanghai, China). ELISA kits for mouse IL-10, IL-6, IL-1β, and TNF-α were purchased from Nanjing Jiancheng Bioengineering Institute (Nanjing, China). Antibodies against CD86 (Pacific Blue™-conjugated), CD206 (APC-conjugated), and F4/80 (PE-conjugated) for flow cytometry were purchased from Biolegend (San Diego, CA, USA).

### Preparation and characterization of Dex@mPLGA

Dex-loaded PLGA nanoparticles (Dex@PLGA) were prepared using the emulsion solvent evaporation method. Briefly, 50 mg of PLGA and 2 mg of Dex were dissolved in 4 mL of dichloromethane. The organic phase was added to 20 mL of 1% (w/v) PVA aqueous solution and sonicated (100 W, 10 min, ice bath) to form a primary emulsion. The emulsion was then stirred at 300 rpm for 8 h to evaporate the organic solvent. The resulting nanoparticles were collected by centrifugation (12,000 rpm, 4 °C, 30 min), washed three times with distilled water, and resuspended in PBS.

To prepare macrophage membrane-coated Dex@PLGA (Dex@mPLGA), RAW264.7 macrophage membranes were isolated as previously described (Zhang et al., 2025). Briefly, RAW264.7 cells were lysed using a membrane protein extraction kit, and the collected membranes were sonicated and extruded through 400 nm and 200 nm polycarbonate membranes. The preformed Dex@PLGA nanoparticles were mixed with the macrophage membranes at a protein to polymer weight ratio of 1:10, followed by sonication (100 W, 2 min, ice bath) to obtain Dex@mPLGA. Following membrane coating, Dex@mPLGA nanoparticles were further purified by centrifugation at 12,000 rpm for 30 min at 4 °C and washed three times with PBS to remove excess free membrane vesicles and loosely associated membrane fragments. FITC-labeled nanoparticles were prepared by adding 1 mg of FITC to the organic phase during the preparation process.

The morphology of Dex@mPLGA was observed using TEM after negative staining with 1% uranyl acetate. The hydrodynamic diameter, PDI, and zeta potential were measured by DLS using a Zetasizer Nano ZS (Malvern, UK). The drug loading content (LC%) and encapsulation efficiency (EE%) were determined by HPLC. Briefly, 4 mg of freeze-dried nanoparticles were dissolved in 1 mL of DMSO, and the Dex concentration was analyzed using an Agilent 1260 Infinity HPLC system with a C18 column. LC% and EE% were calculated according to formulas.

$$\text{Loading Content }\left(\text{LC}\%\right)=\frac{\text{Mass}\;\text{of}\;\text{loaded}\;\text{SOD}}{\text{Total}\;\text{mass}\;\text{of}\;\text{nanoparticles}}\times 100$$

$$\text{Encapsulation Efficiency }\left(\text{EE}\%\right)=\frac{\text{Mass}\;\text{of}\;\text{loaded}\;\text{SOD}}{\text{Initial}\;\text{mass}\;\text{of}\;\text{SOD}\;\text{added}}\times 100$$

For stability assessment, Dex@mPLGA was dispersed in DMEM, 0.9% NaCl, PBS, and ultrapure water, and the particle size was monitored at days 1, 3, 5, and 7 using DLS. For *in vitro* release studies, free Dex, Dex@PLGA, or Dex@mPLGA (equivalent to 1 mg Dex) were placed in dialysis bags (MWCO 14 kDa) and immersed in 200 mL of PBS (pH 7.4) containing 0.2% Tween 80 at 37 °C with shaking. At predetermined time points (0, 2, 4, 6, 12, 24, 36 h), 1 mL of release medium was withdrawn and replaced with fresh medium. The Dex concentration was measured by HPLC. The experiment was independently repeated three times (n = 3), and the data are presented as mean ± SD.

### Cell culture

The RAW264.7 murine macrophage cell line was obtained from the Chinese Academy of Sciences (Shanghai, China). Cells were cultured in DMEM supplemented with 10% FBS at 37 °C in a 5% CO_2_ humidified atmosphere. For LPS stimulation, cells were treated with 100 ng/mL LPS for 24 h unless otherwise specified.

### *In vitro* functional assays

For *in vitro* inflammatory stimulation, RAW264.7 cells were seeded in appropriate culture plates and allowed to adhere overnight. For all *in vitro* experiments, Dex@PLGA and Dex@mPLGA were administered at a nanoparticle concentration of 10 μg/mL, corresponding to an equivalent Dex concentration of approximately 0.32 μg/mL. Cells were subsequently pretreated with free Dex, Dex@PLGA, or Dex@mPLGA at equivalent Dex concentrations for 4 h, followed by stimulation with lipopolysaccharide (LPS, 100 ng/mL) for an additional 24 h. Untreated cells served as the control group.

For cell viability analysis, RAW264.7 cells were seeded into 96-well plates at a density of 5 × 10^3^ cells per well. After the indicated treatments, cell viability was evaluated using the CCK-8 assay according to the manufacturer instructions.

For intracellular ROS detection, cells were incubated with DCFH-DA fluorescent probe after treatment, and fluorescence intensity was analyzed by fluorescence microscopy and microplate reader.

For cytokine secretion analysis, the cell culture supernatants were collected after LPS stimulation for 24 h, and the levels of TNF-α, IL-6, IL-1β, and IL-10 were determined using ELISA kits according to the manufacturers protocols.

For MDA and NO assays, intracellular MDA levels and supernatant NO production were measured using commercial assay kits following the manufacturer instructions.

For apoptosis analysis, treated cells were harvested and stained using Annexin V-FITC/PI apoptosis detection kits, followed by flow cytometric analysis.

For EdU proliferation assays, cells were incubated with EdU reagent after the indicated treatments and analyzed according to the manufacturer protocol.

For macrophage polarization studies, RAW264.7 cells were treated as described above and stained with corresponding antibodies against M1 and M2 phenotype markers, followed by flow cytometric analysis. Data were obtained from three independent biological replicates, and at least 10,000 cell events were collected for each sample.

All experiments involving free Dex, Dex@PLGA, and Dex@mPLGA were performed using equivalent Dex concentrations to ensure comparability among treatment groups, and each experiment was independently repeated three times.

### Animals

Male C57BL/6J mice (6–8 weeks old, body weight 20–25 g) were purchased from SPF (Beijing) Biotechnology Co., Ltd. (Beijing, China). All animal experiments were approved by the Guidelines of Animal Experiments from the Committee of Medical Ethics (Animal usage license number: 20260401788316) and were performed in accordance with the National Institutes of Health guidelines for the care and use of laboratory animals. Mice were housed under specific pathogen-free conditions with a 12 h light/dark cycle, constant temperature (22 °C–24 °C), and humidity (50%–70%), with free access to food and water.

For the ALI model, mice were anesthetized with isoflurane and received intranasal instillation of LPS (10 mg/kg in 50 μL of sterile saline) as previously described (Wang et al., 2024). Control mice received an equal volume of saline. After model establishment, mice were randomly allocated to different experimental groups using a random number table. For *in vivo* biodistribution and efficacy studies, 24 h after LPS challenge, mice were intravenously injected via the tail vein with free Dex, Dex@PLGA, or Dex@mPLGA at a Dex-equivalent dose of 5 mg/kg. This single-dose regimen was used for the survival cohort, serum cytokine cohort, lung histology cohort, and biodistribution cohort. At various time points (4, 8, 12, 24 h post-injection), mice were euthanized, and major organs (heart, liver, spleen, lung, kidneys) were collected for *ex vivo* fluorescence imaging using an IVIS Lumina XR system (PerkinElmer, USA). For biodistribution analysis, n = 3 mice per group at each time point. For survival analysis, mice were monitored for 96 h, and survival curves were plotted using the Kaplan-Meier method, with n = 10 mice per group. For histological and biochemical analyses, mice were euthanized 24 h after treatment, and blood samples were collected for serum isolation. Lung tissues were fixed in 4% paraformaldehyde for H&E staining, n = 5 mice per group. To quantitatively assess lung injury, H&E-stained lung sections were evaluated using a blinded semi-quantitative lung-injury scoring method based on standardized histopathological criteria with minor modifications (Jiang et al., 2020). Four pathological features were assessed: inflammatory-cell infiltration, hemorrhage, and edema. Each parameter was scored from 0 to 4 according to injury severity: 0, absent; 1, mild; 2, moderate; 3, severe; and 4, very severe. Five randomly selected fields from each lung section were analyzed for each mouse. The total lung-injury score was calculated as the sum of the individual parameter scores, with higher scores indicating more severe lung injury. Lung histopathological image analysis and lung-injury scoring were independently performed by two investigators who were blinded to group allocation, and the final scores were expressed as mean ± SD for statistical analysis.

For biosafety evaluation, healthy mice were intravenously injected with Dex@mPLGA daily for 7 days. Control mice received saline. On day 8, mice were euthanized, and major organs were collected for H&E staining. Serum levels of alanine aminotransferase (ALT), aspartate aminotransferase (AST), blood urea nitrogen (BUN), creatinine (CR), and uric acid (UA) were measured using commercial kits (Nanjing Jiancheng, China).

At the study endpoint, mice were euthanized using a CO_2_ chamber with gradual-fill CO_2_ at a flow rate of 30% chamber volume/min. When lung tissues and other biological samples were collected, death of the mice had been confirmed.

### Statistical analysis

All experiments were performed at least three independent times, and data are presented as mean ± SD. Statistical analyses were conducted using GraphPad Prism 8.0 software (GraphPad Software, San Diego, CA, USA). Comparisons between two groups were performed using the unpaired two-tailed Student’s t-test. For comparisons involving three or more groups, one-way analysis of variance (ANOVA) followed by Tukey’s *post hoc* test was applied. Survival curves were analyzed using the Kaplan-Meier method with the log-rank (Mantel–Cox) test. A p value of less than 0.05 was considered statistically significant. Significance levels are indicated as *p < 0.05, **p < 0.01, and ***p < 0.001.

## Results and discussion

### Preparation and characterization of Dex@mPLGA

**FIGURE 1:**
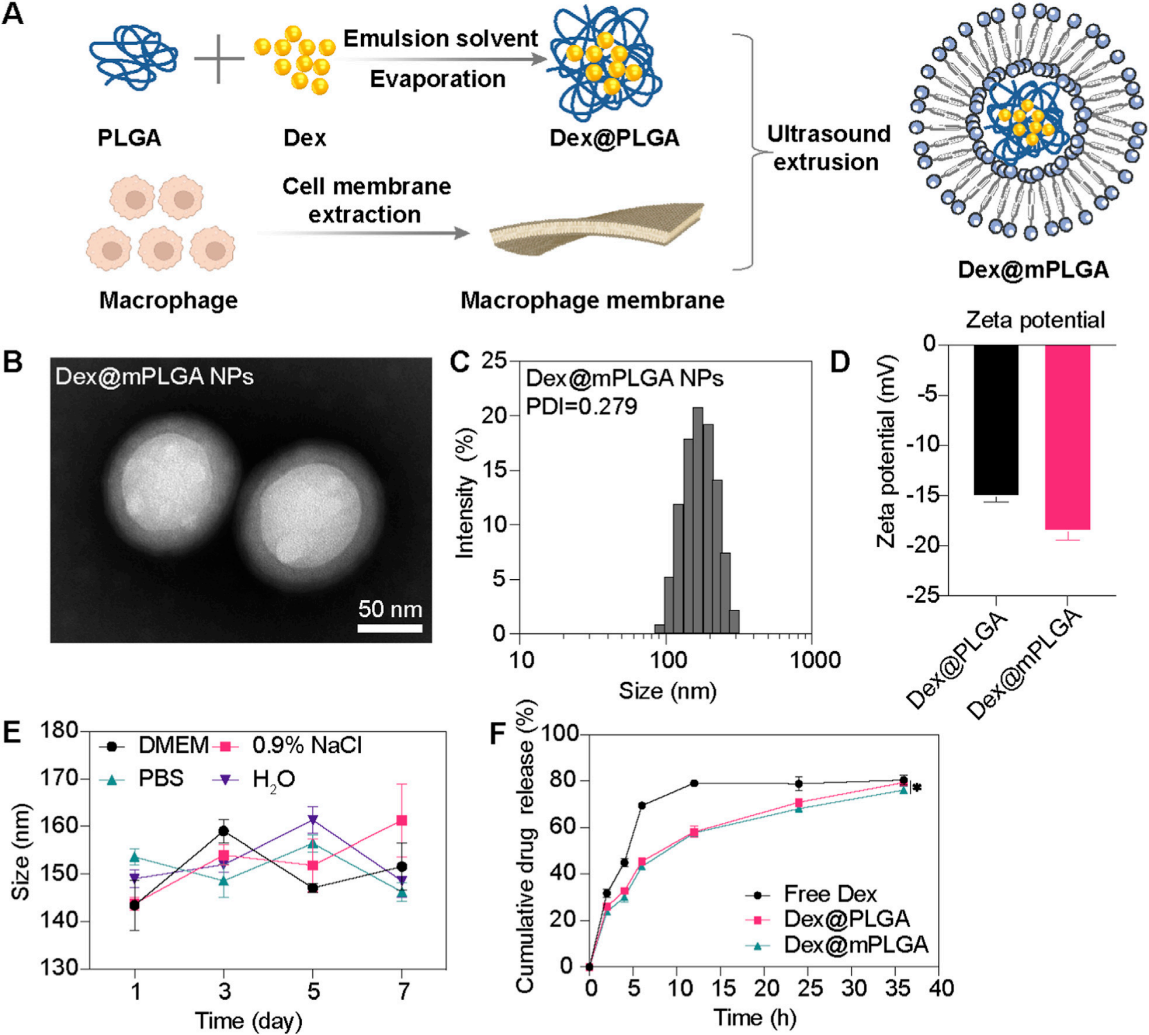
Preparation and Characterization of Dex@mPLGA. **(A)** Schematic diagram of the synthesis route for Dex@mPLGA nanoparticles. **(B)** TEM image of Dex@mPLGA. Scale bar = 50 nm. **(C)** Size distribution profile of Dex@mPLGA. **(D)** Zeta potentials of Dex@PLGA and Dex@mPLGA. **(E)** Storage stability evaluation of Dex@mPLGA in DMEM, 0.9% NaCl, PBS, and water, assessed by measuring particle size changes on days 1, 3, 5, and 7. **(F)** *In vitro* cumulative drug release profiles of free dexamethasone (Free Dex), Dex@PLGA, and Dex@mPLGA. Data are presented as mean ± SD. *p < 0.05.

Dexamethasone-loaded PLGA nanoparticles (Dex@PLGA) were successfully fabricated via an emulsion solvent evaporation method, followed by cloaking with macrophage membranes to obtain the biomimetic delivery system Dex@mPLGA. As shown in Supplementary Figure S1, Dex@PLGA nanoparticles were successfully prepared, exhibiting a well-defined spherical morphology with uniform size distribution, confirming the successful formation of the PLGA-based drug-loaded core structure prior to membrane coating. The preparation workflow is depicted in Figure 1A. TEM analysis confirmed a typical core–shell architecture, with a continuous and uniform membrane layer enveloping the nanoparticle surface, demonstrating the successful incorporation of macrophage membranes (Figure 1B). DLS measurements indicated that Dex@mPLGA possessed an average hydrodynamic diameter of 147.3 nm and a PDI of 0.279, reflecting a favorable size distribution and colloidal stability (Figure 1C). Moreover, Dex@mPLGA exhibited a more negative zeta potential compared to uncoated Dex@PLGA (Figure 1D), further validating successful membrane coating. As shown in Supplementary Figure S2, SDS-PAGE analysis was performed to further evaluate the retention of RAW264.7 membrane proteins in Dex@mPLGA. Dex@mPLGA displayed multiple protein bands that overlapped with those of the cell membrane lysate, whereas uncoated Dex@PLGA showed no obvious protein bands, supporting the retention of RAW264.7 membrane protein components in the final membrane-coated formulation.

The drug loading capacity and encapsulation efficiency of Dex@mPLGA were quantified by HPLC, revealing a loading capacity of 3.2% and an encapsulation efficiency of 74.6%. To assess colloidal stability, Dex@mPLGA was suspended in DMEM, 0.9% NaCl, PBS, and ultrapure water, and particle size changes were monitored on days 1, 3, 5, and 7. As shown in Figure 1E, no significant particle size increase was detected in any medium, indicating that Dex@mPLGA exhibits excellent storage and dispersion stability.

The *in vitro* drug release profiles were subsequently evaluated. Compared with free dexamethasone, both Dex@PLGA and Dex@mPLGA demonstrated sustained release behavior, with Dex@mPLGA displaying the slowest release rate (Figure 1F). Compared with free Dex, both Dex@PLGA and Dex@mPLGA exhibited sustained release behavior. Although their overall release trends were similar, Dex@mPLGA showed significantly lower cumulative release than Dex@PLGA at later time points, suggesting that macrophage membrane coating may moderately retard Dex diffusion by acting as an additional diffusion barrier. Overall, Dex@mPLGA features a well-defined core–shell structure, robust physicochemical stability, and enhanced sustained-release performance, establishing a promising platform for efficient anti-inflammatory drug delivery.

Cellular Uptake and Biocompatibility Effects of Dex@mPLGA.

**FIGURE 2:**
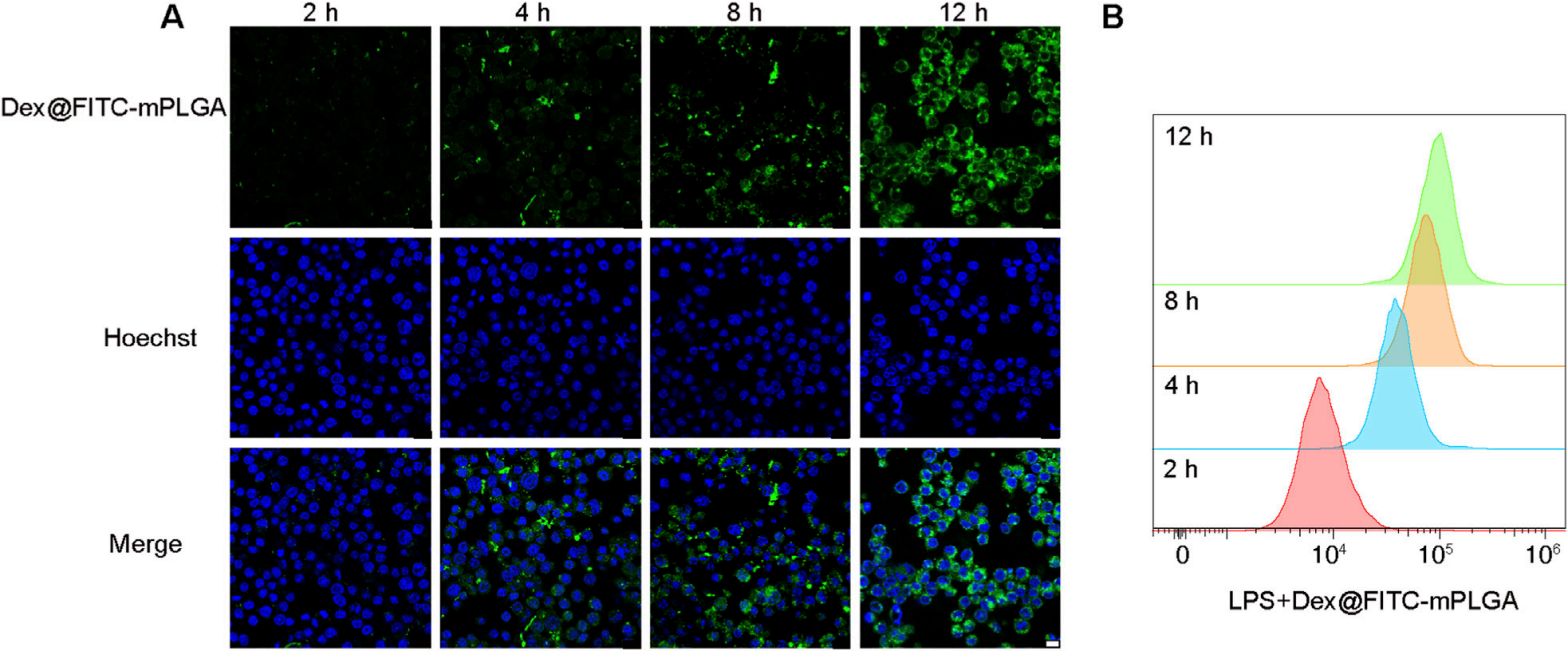
Cellular uptake of FITC-labeled Dex@mPLGA by RAW264.7. **(A)** Representative CLSM images showing the time-dependent internalization of Dex@mPLGA after 2, 4, 8, and 12 h of incubation. Scale bar = 10 μm. **(B)** Flow cytometric histograms demonstrating the progressive increase in fluorescence intensity over time, indicating efficient and time-dependent cellular uptake of Dex@mPLGA (n = 3).

First, the cellular internalization of Dex@mPLGA was evaluated in RAW264.7 macrophages. After incubation with FITC-labeled Dex@mPLGA for 2, 4, 8, and 12 h, CLSM imaging revealed a progressive, time-dependent increase in intracellular green fluorescence intensity (Figure 2A). As shown in Supplementary Figure S3, both FITC-labeled Dex@PLGA and Dex@mPLGA nanoparticles were effectively internalized by RAW264.7 macrophages in a time-dependent manner. Notably, Dex@mPLGA exhibited significantly stronger intracellular fluorescence intensity compared with uncoated Dex@PLGA, particularly after prolonged incubation. These results indicate that macrophage membrane coating enhances nanoparticle uptake by macrophages, likely through improved homologous membrane interaction and enhanced macrophage-recognition capability.

Consistently, flow cytometric analysis showed a gradual rightward shift of the fluorescence peak accompanied by continuously elevated fluorescence intensity (Figure 2B), confirming efficient and time-dependent macrophage uptake of Dex@mPLGA. These observations align with previous reports demonstrating that homotypic cell membrane–camouflaged biomimetic nanoplatforms exhibit enhanced cellular internalization efficiency (Jiménez-Jiménez et al., 2023). Prior to assessing anti-inflammatory activity, the biosafety of the nanoparticles was examined. As shown in Supplementary Figure S4, RAW264.7 cell viability remained above 80% after 24 h of treatment with Dex@mPLGA at concentrations up to 16 μM, indicating favorable biocompatibility and negligible cytotoxicity within the therapeutic range.

**FIGURE 3:**
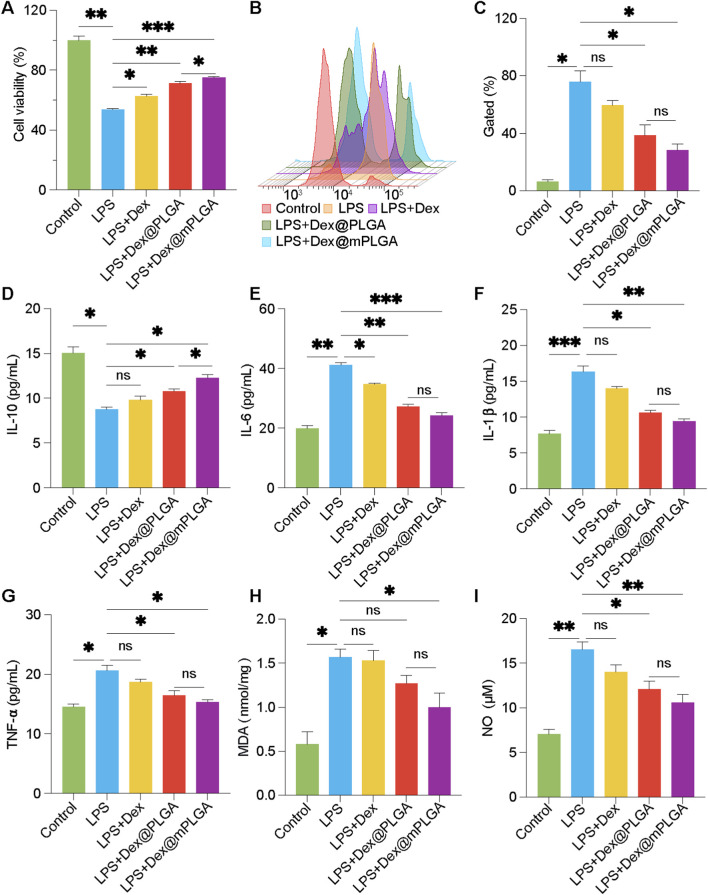
Cytoprotective and anti-inflammatory effects of Dex@mPLGA in LPS-stimulated RAW264.7 cells. **(A)** Cell viability determined by CCK-8 assay after treatment with free Dex, Dex@PLGA, or Dex@mPLGA. **(B,C)** Representative flow cytometric histograms **(B)** and quantitative analysis **(C)** of intracellular reactive oxygen species (ROS) levels. **(D–G)** Secretion levels of anti-inflammatory cytokine IL-10 **(D)** and pro-inflammatory cytokines IL-6 **(E)**, IL-1β **(F)**, and TNF-α **(G)** measured by ELISA. **(H)** Intracellular malondialdehyde (MDA) levels detected using a commercial kit. **(I)** Nitric oxide (NO) levels in the culture supernatant. Data are presented as mean ± SD (n = 3). *p < 0.05, **p < 0.01, ***p < 0.001.

The cytoprotective efficacy of Dex@mPLGA was next assessed using an LPS-induced macrophage inflammation model. As shown in Figure 3A, LPS stimulation markedly reduced cell viability, while Dex@mPLGA significantly restored cellular survival. Notably, its cytoprotective capacity surpassed that of both free Dex and Dex@PLGA, demonstrating that the biomimetic nanodelivery system enhances the anti-inflammatory efficacy of dexamethasone. Considering the critical role of oxidative stress in LPS-induced injury, intracellular ROS levels were quantified. Flow cytometric analyses (Figures 3B,C) demonstrated that Dex@mPLGA markedly inhibited LPS-induced ROS overproduction, exhibiting a substantially stronger antioxidant effect. These results collectively confirm the potent antioxidant activity of Dex@mPLGA, likely attributable to its enhanced intracellular accumulation and sustained drug retention enabled by the biomimetic design.

To further elucidate its immunomodulatory function, the cytokine secretion profile was assessed. As shown in Figures 3D–G, Dex@mPLGA significantly increased the production of the anti-inflammatory cytokine IL-10, while markedly suppressing pro-inflammatory cytokines including IL-6, IL-1β, and TNF-α. The magnitude of these regulatory effects exceeded that of free Dex and Dex@PLGA, highlighting the augmented immunomodulatory performance offered by the biomimetic formulation. Correspondingly, MDA, a hallmark product of lipid peroxidation, was significantly reduced following Dex@mPLGA treatment (Figure 3H). In parallel, NO production was quantified. As illustrated in Figure 3I, LPS stimulation induced substantial NO elevation, which was dramatically attenuated by Dex@mPLGA. Given that NO overproduction, catalyzed by inducible nitric oxide synthase (iNOS), is a prominent contributor to inflammation-mediated tissue damage, this finding further underscores the anti-inflammatory potency of Dex@mPLGA. Together, these data demonstrate that Dex@mPLGA effectively reshapes the inflammatory microenvironment by attenuating oxidative stress, downregulating pro-inflammatory cytokines and NO, and enhancing IL-10 secretion. Through these combined actions, Dex@mPLGA confers pronounced cytoprotective effects in LPS-activated macrophages, supporting its promise as a therapeutic nanoplatform for inflammatory disorders.

**FIGURE 4:**
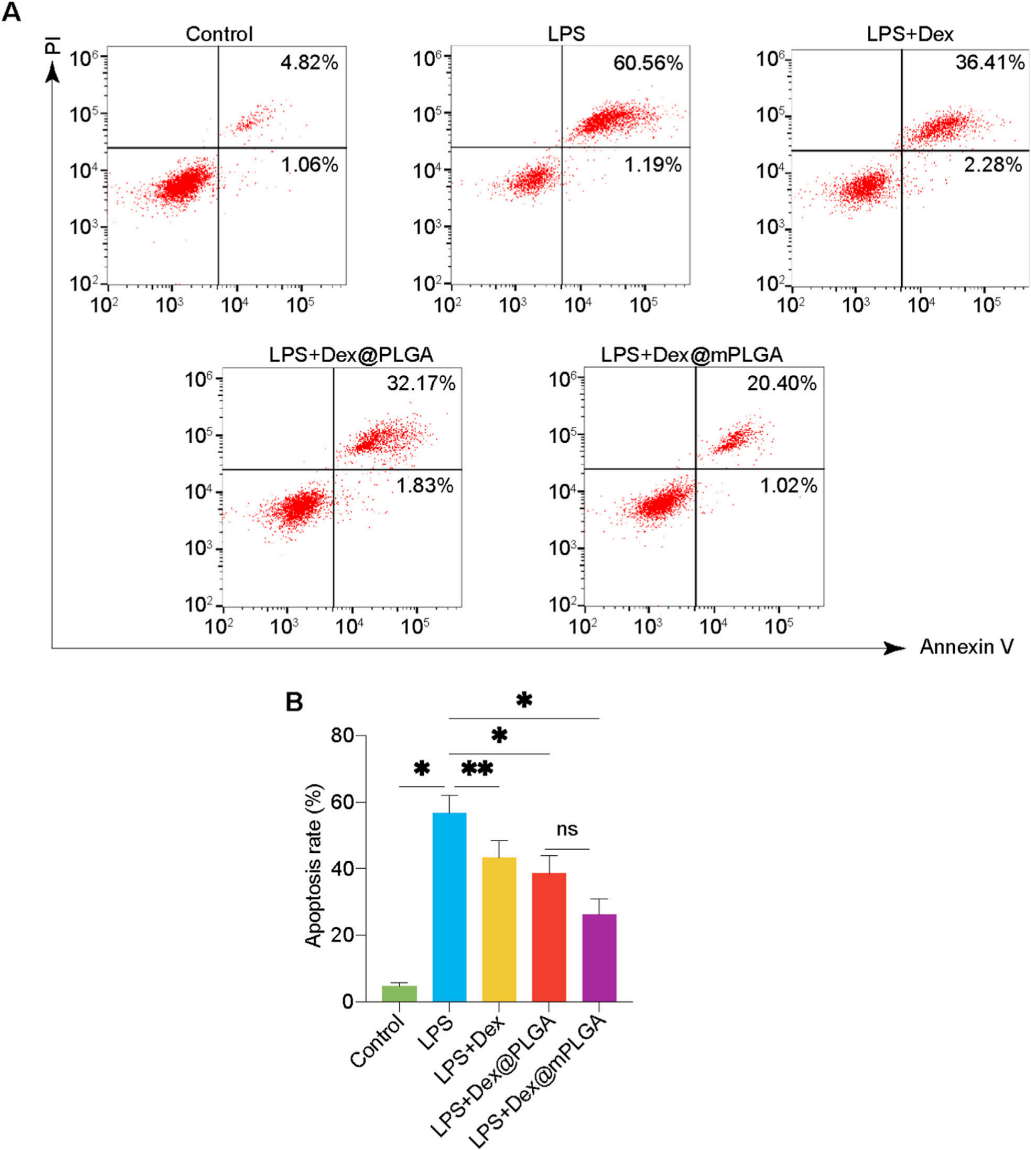
Effect of Dex@mPLGA on LPS-induced apoptosis in RAW264.7 cells. **(A)** Representative flow cytometric plots of Annexin V-FITC/PI double staining. **(B)** Quantitative analysis of the total apoptotic rate (early and late apoptosis). Data are presented as mean ± SD (n = 3). *p < 0.05, **p < 0.01, ***p < 0.001.

To further corroborate its cytoprotective function, apoptosis in LPS-stimulated RAW264.7 cells was evaluated by flow cytometry. As shown in Figure 4A, LPS treatment markedly increased macrophage apoptosis to 56.61% ± 5.49%. Free Dex reduced the apoptotic rate to 43.37% ± 5.08%, and Dex@PLGA further decreased it to 38.60% ± 5.46%. Importantly, Dex@mPLGA elicited a substantially greater reduction, lowering apoptosis to 26.26% ± 4.60% (Figure 4B), demonstrating superior anti-apoptotic performance relative to free Dex and Dex@PLGA. These findings suggest that enhanced cellular uptake and sustained anti-inflammatory activity contribute to the potent anti-apoptotic efficacy of the biomimetic nanoformulation.

**FIGURE 5:**
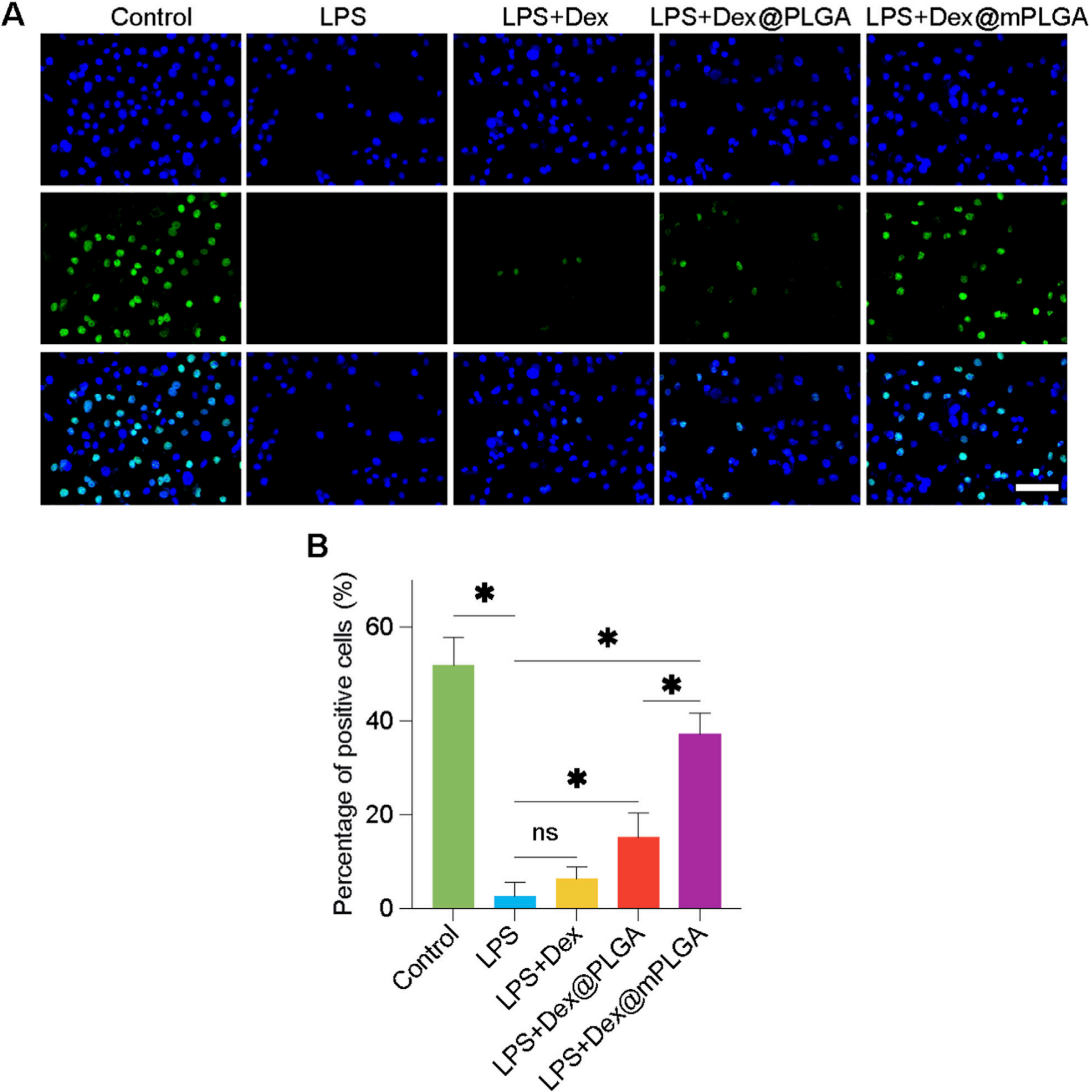
Effect of Dex@mPLGA on LPS-inhibited cell proliferation in RAW264.7 cells assessed by EdU incorporation assay. **(A)** Representative fluorescence microscopy images of EdU-stained cells (green) and Hoechst (blue). Scale bar = 100 μm. **(B)** Quantitative analysis of EdU-positive cells. Data are presented as mean ± SD (n = 3). *p < 0.05, **p < 0.01, ***p < 0.001.

The effects of Dex@mPLGA on LPS-impaired proliferation were further investigated using an EdU incorporation assay. Representative images (Figure 5A) and quantitative analysis (Figure 5B) showed that LPS drastically reduced the proportion of EdU-positive cells to 2.68%, indicating severe proliferation arrest. Free Dex and Dex@PLGA partially rescued proliferation, increasing EdU-positive cells to 6.32% and 15.16%, respectively. In contrast, Dex@mPLGA substantially restored proliferative activity to 37.24%, significantly outperforming both free Dex and Dex@PLGA. These data further confirm the strong cytoprotective capacity of Dex@mPLGA in reversing LPS-induced proliferative suppression.

Collectively, the apoptosis and proliferation results demonstrate that Dex@mPLGA markedly enhances macrophage survival under inflammatory stress by simultaneously inhibiting apoptosis and promoting proliferation. These findings provide compelling evidence supporting Dex@mPLGA as a promising therapeutic strategy for inflammatory diseases. Taken together, Dex@mPLGA and Dex@PLGA showed comparable effects in most inflammatory and oxidative stress-related *in vitro* endpoints, while Dex@mPLGA exhibited significant advantages in selected readouts, including cell viability, IL-10 secretion, EdU-positive proliferation, and M2 polarization, suggesting that macrophage membrane coating may confer selective rather than universal biological benefits that require further validation in more physiologically relevant models.

### *In vitro* anti-inflammatory modulation of Dex@mPLGA

**FIGURE 6:**
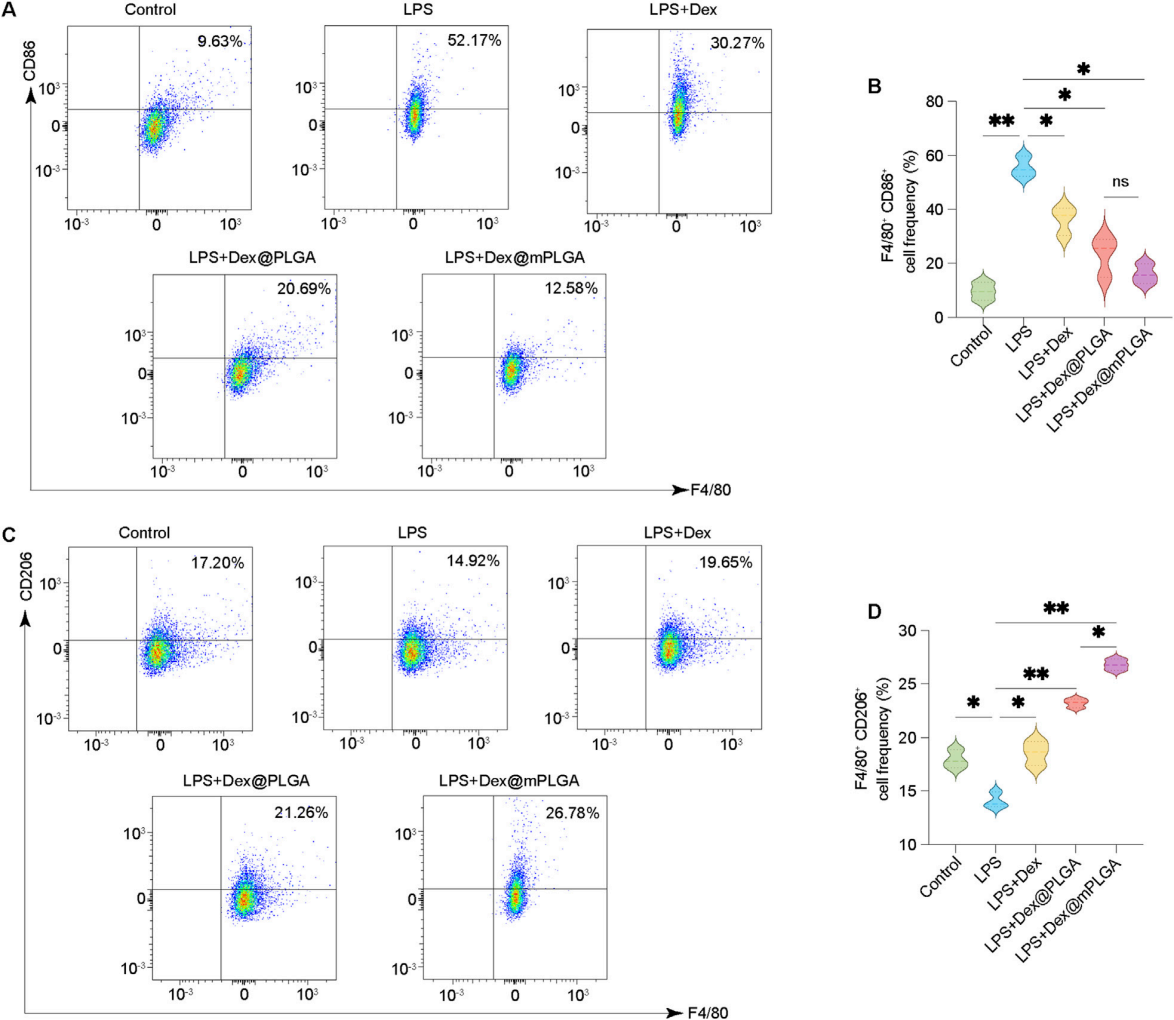
Modulation of macrophage polarization by Dex@mPLGA in LPS-stimulated RAW264.7 cells. **(A)** Representative flow cytometric plots of CD86 and F4/80 co-staining. **(B)** Quantitative analysis of CD86+F4/80+ cell percentages (M1-type macrophages). **(C)** Representative flow cytometric plots of CD206 and F4/80 co-staining. **(D)** Quantitative analysis of CD206+F4/80+ cell percentages (M2-type macrophages). Data are presented as mean ± SD (n = 3). *p < 0.05, **p < 0.01, ***p < 0.001.

To precisely assess the impact of Dex@mPLGA on macrophage polarization, flow cytometry was used to quantify CD86+F4/80+ double-positive cells (M1 phenotype) and CD206+F4/80+ double-positive cells (M2 phenotype). As shown in Figures 6A,B, LPS stimulation markedly increased the proportion of CD86+F4/80+ cells, indicating a shift toward the pro-inflammatory M1 phenotype. Treatment with free Dex or Dex@PLGA partially mitigated this increase, whereas Dex@mPLGA produced the most significant reduction, demonstrating its superior ability to inhibit M1 polarization. Conversely, LPS exposure substantially decreased the proportion of CD206+F4/80+ cells, reflecting suppressed M2 polarization (Figures 6C,D). Dex@mPLGA treatment effectively restored CD206+F4/80+ cell levels, exhibiting a markedly stronger effect than free Dex or Dex@PLGA. Overall, these results indicate that Dex@mPLGA simultaneously suppresses LPS-induced M1 polarization and promotes repolarization toward the M2 phenotype, thereby contributing to the restoration of immune homeostasis within the inflammatory microenvironment.

### *In vivo* anti-inflammatory efficacy and biosafety of Dex@mPLGA

**FIGURE 7:**
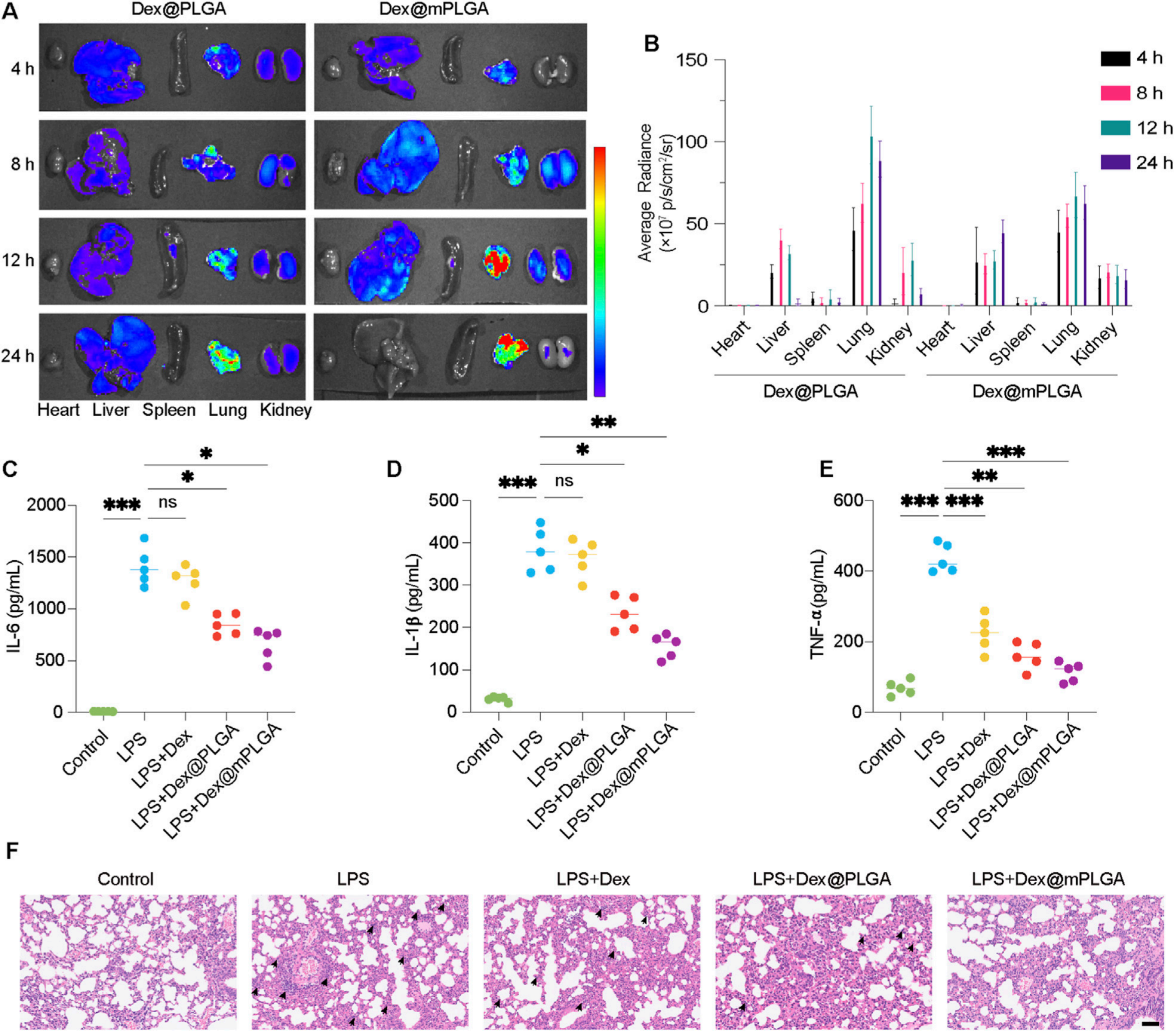
*In vivo* anti-inflammatory efficacy of Dex@PLGA and Dex@mPLGA in a mouse model of LPS-induced acute lung injury (ALI). **(A)** *Ex vivo* fluorescence imaging of major organs (heart, liver, spleen, lung, kidney) at 4, 8, 12, and 24 h after intravenous injection of Dex@mPLGA (n = 3). **(B)** Quantitative analysis of fluorescence intensity in each organ over time (n = 3). **(C–E)** Serum levels of pro-inflammatory cytokines IL-6 **(C)**, IL-1β **(D)**, and TNF-α **(E)** measured by ELISA (n = 5). **(F)** Representative H&E-stained lung sections; black arrows indicate inflammatory cell infiltration (n = 5). Scale bar = 50 μm. Data are presented as mean ± SD. *p < 0.05, **p < 0.01, ***p < 0.001.

To further assess the *in vivo* anti-inflammatory efficacy of Dex@mPLGA, an acute lung injury (ALI) mouse model was induced by intranasal administration of LPS. At 24 h post-induction, Dex@PLGA and Dex@mPLGA was administered via tail vein injection, and nanoparticle biodistribution was tracked at 4, 8, 12, and 24 h. As shown in Figures 7A,B, nanoparticles primarily accumulated in the liver and kidneys at the early stage. With prolonged circulation, fluorescence signals progressively increased in the lung and remained detectable up to 24 h, indicating effective nanoparticle accumulation at the inflammatory site. This targeted enrichment is likely driven by the macrophage membrane coating, which confers inflammation-homing capability and facilitates preferential uptake by activated pulmonary macrophages.

Survival analysis (Supplementary Figure S5) revealed that all mice in the LPS model group succumbed within 72 h. In comparison, free Dex and Dex@PLGA treatments improved survival to 20% and 40%, respectively, whereas Dex@mPLGA achieved a significantly enhanced survival rate of 80%. Correspondingly, serum cytokine measurements (Figures 7C–E) demonstrated that Dex@mPLGA markedly suppressed the LPS-induced elevation of pro-inflammatory cytokines TNF-α, IL-6, and IL-1β, outperforming both free Dex and Dex@PLGA. Histopathological evaluation of lung tissues by H&E staining (Figure 7F) further supported these findings. The LPS group exhibited pronounced alveolar wall thickening and extensive inflammatory cell infiltration, whereas Dex@mPLGA treatment substantially ameliorated these pathological abnormalities, restoring lung tissue morphology toward normal. To further support the histological observations, semiquantitative lung-injury scoring was performed based on H&E-stained lung tissue sections. As shown in Supplementary Figure S6, the LPS group exhibited a markedly increased lung-injury score, indicating severe pulmonary pathological damage. Treatment with Dex@PLGA or Dex@mPLGA reduced the lung-injury score to varying degrees, suggesting that Dex-loaded nanoparticle formulations alleviated LPS-induced lung injury. The scoring analysis was based on inflammatory-cell infiltration, hemorrhage, and edema across the indicated experimental groups (n = 5), providing quantitative support for the representative H&E images. Collectively, these data validate the potent *in vivo* anti-inflammatory activity of Dex@mPLGA and highlight its therapeutic potential for ALI.

Regarding biosafety, H&E staining of major organs (heart, liver, spleen, lung, and kidney) after 7 days of continuous administration revealed no discernible pathological abnormalities in the Dex@mPLGA group compared with controls (Supplementary Figure S7A). Additionally, serum biochemical indicators, including ALT, AST, BUN, CR, and UA, remained within normal physiological ranges and showed no significant differences between Dex@mPLGA-treated and untreated groups (Supplementary Figures S7B–F). These results collectively confirm the favorable *in vivo* biosafety profile of Dex@mPLGA, supporting its suitability for further translational development.

## Discussion

In the present study, we developed a biomimetic macrophage membrane-coated dexamethasone delivery platform, Dex@mPLGA, for the treatment of sepsis-induced acute lung injury. Compared with free Dex, Dex@mPLGA showed broad anti-inflammatory, antioxidant, cytoprotective, and immunomodulatory effects. However, when directly compared with uncoated Dex@PLGA, the incremental *in vitro* benefit of macrophage membrane coating was endpoint-dependent rather than uniform. Direct Dex@PLGA controls allowed us to distinguish the contribution of the PLGA core from that of membrane coating. These comparisons showed statistically significant coating-related improvement only in selected *in vitro* endpoints, including cell viability, IL-10 production, EdU proliferation, and M2 polarization.

One important concern was whether the therapeutic performance of Dex@mPLGA could simply result from differences in Dex release kinetics or extracellular drug availability rather than from the biomimetic membrane coating itself. To minimize this potential confounding factor, all experimental groups in the present study were normalized based on equivalent Dex concentrations calculated from the measured drug loading capacity. Therefore, the observed biological differences among free Dex, Dex@PLGA, and Dex@mPLGA cannot be attributed to unequal nominal drug dosing. The release analysis showed that both Dex@PLGA and Dex@mPLGA exhibited sustained release behavior compared with free Dex. Although their overall release profiles were generally similar, Dex@mPLGA showed a modestly delayed cumulative release at later time points, suggesting that macrophage membrane coating may slightly retard Dex diffusion from the PLGA core. Nevertheless, this release difference should be interpreted cautiously and should not be regarded as the major mechanism underlying the biological performance of Dex@mPLGA.

The uptake comparison experiments showed increased cellular internalization of Dex@mPLGA compared with Dex@PLGA in RAW264.7 macrophages, supporting a possible role of membrane coating in macrophage-associated uptake. However, this interpretation should be made with caution. In the present study, RAW264.7 cells were used as the macrophage membrane source, the *in vitro* uptake target, and the LPS-induced inflammatory macrophage model. Although this design is useful for evaluating nanoparticle uptake and anti-inflammatory activity in a macrophage-associated inflammatory setting, it cannot fully distinguish macrophage membrane-specific targeting from the intrinsic phagocytic uptake of coated nanoparticles by macrophages. Therefore, the enhanced uptake observed in RAW264.7 cells should be interpreted as evidence of increased macrophage-associated internalization rather than definitive proof of a specific homotypic-targeting mechanism. The extent to which macrophage membrane coating confers membrane-specific targeting beyond generic phagocytosis will require further validation using non-homologous membrane controls, primary macrophages, and *in vivo* alveolar macrophage populations.

Following internalization, the PLGA core may serve as an intracellular Dex reservoir and contribute to sustained local Dex exposure. However, because real-time intracellular and extracellular Dex concentrations were not directly quantified, the relative contributions of enhanced uptake, intracellular retention, and release kinetics remain to be further clarified. Statistically significant advantages of Dex@mPLGA over Dex@PLGA were observed mainly for cell viability, IL-10 secretion, EdU proliferation, and M2 polarization. For several inflammatory and oxidative stress-related readouts, including ROS, IL-6, IL-1β, TNF-α, MDA, NO, apoptosis, and M1 polarization, Dex@PLGA and Dex@mPLGA showed comparable effects. These results indicate that the membrane coating provided selected *in vitro* benefits rather than a universal enhancement across all endpoints.

The biodistribution analysis comparing Dex@PLGA and Dex@mPLGA suggested improved pulmonary accumulation of Dex@mPLGA in the ALI model, although fluorescence imaging alone cannot fully establish intact nanoparticle targeting or define the underlying mechanism. These delivery-related properties may contribute to the improved *in vivo* therapeutic outcomes observed for Dex@mPLGA. Compared with the LPS model, Dex@mPLGA reduced systemic inflammatory cytokine levels, alleviated histopathological lung injury, and improved survival in septic ALI mice. Nevertheless, the present data do not allow us to attribute these *in vivo* benefits exclusively to macrophage membrane-specific targeting, because passive nanoparticle accumulation, macrophage phagocytosis, PLGA-mediated sustained drug delivery, and membrane-associated interactions may all contribute to the overall therapeutic effect.

The successful purification and characterization of Dex@mPLGA support the feasibility of this biomimetic nanoplatform. Following membrane coating, excess free membrane vesicles and loosely associated membrane fragments were removed by centrifugation and washing procedures before biological evaluation. TEM imaging revealed a characteristic core–shell morphology, while particle size distribution and zeta-potential analyses supported membrane incorporation and relatively homogeneous nanoparticle formation. In addition, SDS-PAGE analysis provided protein-level supportive evidence for the retention of macrophage membrane protein components in Dex@mPLGA.

In addition, while the current characterization data support successful membrane coating, more advanced approaches such as density-gradient purification, membrane protein profiling, fluorescence co-localization analysis, FRET-based membrane integrity assays, nano-flow cytometry, or nanoparticle tracking analysis would further strengthen validation of coating integrity and nanoparticle homogeneity. Future studies should also incorporate non-homologous membrane controls and validate nanoparticle uptake in primary macrophages, alveolar macrophages, or *in vivo* pulmonary immune-cell subsets to more rigorously determine whether macrophage membrane coating confers specific targeting beyond generic macrophage phagocytosis.

Taken together, our findings suggest that the therapeutic effects of Dex@mPLGA are likely associated with PLGA-mediated sustained Dex delivery, enhanced macrophage-associated uptake, improved pulmonary accumulation in the inflammatory lung microenvironment, and selected membrane-related immunoregulatory benefits. However, the incremental *in vitro* advantage of Dex@mPLGA over Dex@PLGA was modest and endpoint-dependent, and the current RAW264.7-based design does not definitively establish a specific homotypic-targeting mechanism. The conclusion supports Dex@mPLGA as a promising biomimetic nanoplatform for sepsis-induced ALI while acknowledging that membrane-specific targeting requires further validation in more physiologically relevant models.

## Conclusion

In this study, we developed a biomimetic nanoplatform, Dex@mPLGA, by coating dexamethasone-loaded PLGA nanoparticles with RAW264.7 macrophage membranes and systematically investigated its therapeutic potential for sepsis-induced ALI. Dex@mPLGA exhibited a well-defined core–shell architecture, favorable physicochemical characteristics, including a mean particle size of 147.3 nm, PDI of 0.279, negative zeta potential, drug loading of 3.2%, and encapsulation efficiency of 74.6%. The formulation also showed good colloidal stability and sustained drug-release behavior, supporting its suitability as a stable Dex delivery system. *In vitro* evaluations revealed that Dex@mPLGA was efficiently internalized by RAW264.7 macrophages in a time-dependent manner, with minimal cytotoxicity at concentrations up to 16 μM. In an LPS-induced inflammatory macrophage model, Dex@mPLGA effectively alleviated inflammatory and oxidative stress-related responses relative to the LPS model, as reflected by improved cell viability, reduced ROS and MDA levels, decreased pro-inflammatory cytokines and NO production, increased IL-10 secretion, reduced apoptosis, restored proliferative activity, and modulation of macrophage polarization. Importantly, direct comparison with uncoated Dex@PLGA indicated that the additional benefit of macrophage membrane coating was endpoint-dependent rather than universal. Dex@mPLGA also effectively inhibited LPS-induced apoptosis and restored proliferative capacity, as evidenced by flow cytometry and EdU analyses. Mechanistically, the biomimetic nanocarrier regulated macrophage phenotypic balance by reducing M1 (CD86+F4/80+) polarization and promoting M2 (CD206+F4/80+) polarization, thereby contributing to the reestablishment of immune homeostasis within the inflammatory microenvironment. However, the reduction in M1 polarization was comparable between Dex@PLGA and Dex@mPLGA, indicating that the membrane-coating benefit in macrophage phenotype regulation was selective.

*In vivo*, Dex@mPLGA showed preferential accumulation in inflamed pulmonary tissues in an LPS-induced ALI mouse model, was associated with improved survival outcomes, reduced systemic pro-inflammatory cytokine levels, and alleviated lung histopathological injury, including decreased alveolar wall thickening and inflammatory cell infiltration. These results suggest that macrophage membrane coating may contribute to improved pulmonary delivery and therapeutic performance in the inflammatory lung microenvironment. Meanwhile, repeated-dose biosafety assessment revealed no obvious pathological abnormalities in major organs or significant changes in serum biochemical indices, supporting the preliminary safety profile of this biomimetic nanoplatform.

Collectively, these findings indicate that Dex@mPLGA represents an innovative and effective biomimetic Dex delivery platform for sepsis-induced ALI. Its therapeutic activity is likely associated with the combined contribution of PLGA mediated sustained Dex delivery, macrophage-associated cellular uptake, selected membrane-related immunoregulatory effects, and improved pulmonary accumulation *in vivo*. However, the incremental *in vitro* advantage of Dex@mPLGA over Dex@PLGA was modest and endpoint dependent; therefore, the membrane coating should be interpreted as providing selected auxiliary benefits rather than uniformly enhancing all anti-inflammatory, antioxidant, and anti-apoptotic outcomes.

## Data Availability

The original contributions presented in the study are included in the article/Supplementary Material, further inquiries can be directed to the corresponding authors.

## References

[B1] Abdollahi E. Johnston T. P. Ghaneifar Z. Vahedi P. Goleij P. Azhdari S. (2023). Immunomodulatory therapeutic effects of curcumin on M1/M2 macrophage polarization in inflammatory diseases. Curr. Molecular Pharmacology 16 (1), 2–14. 10.2174/1874467215666220324114624 35331128

[B2] Al-Hetty H. R. A. K. Kadhim M. S. Al-Tamimi J. H. Z. Ahmed N. M. Jalil A. T. Saleh M. M. (2023). Implications of biomimetic nanocarriers in targeted drug delivery. Emergent Mater. 6 (1), 1–13. 10.1007/s42247-023-00453-8 36686331 PMC9846706

[B3] Cao M. Wang G. Xie J. (2023). Immune dysregulation in sepsis: experiences, lessons and perspectives. Cell Death Discovery 9 (1), 465. 10.1038/s41420-023-01766-7 38114466 PMC10730904

[B4] Cheng L. Cao Y. Liu S. Lv L. Zhang J. Bao J. (2025). Unveiling the research advances of sepsis: pathogenesis, precise intervention and clinical perspective. Int. J. Surg. 111 (9), 6260–6289. 10.1097/JS9.0000000000002668 40549442 PMC12430928

[B5] Furnari S. Ciantia R. Garozzo A. Furneri P. M. Fuochi V. (2025). Lactobacilli-Derived microbe-associated molecular patterns (MAMPs) in host immune modulation. Biomolecules 15 (11), 1609. 10.3390/biom15111609 41301527 PMC12650587

[B6] Gorman E. A. O’Kane C. M. McAuley D. F. (2022). Acute respiratory distress syndrome in adults: diagnosis, outcomes, long-term sequelae, and management. Lancet 400 (10358), 1157–1170. 10.1016/S0140-6736(22)01439-8 36070788

[B7] Jiang J. Huang K. Xu S. Garcia J. G. N. Wang C. Cai H. (2020). Targeting NOX4 alleviates sepsis-induced acute lung injury *via* attenuation of redox-sensitive activation of CaMKII/ERK1/2/MLCK and endothelial cell barrier dysfunction. Redox Biology 36, 101638. 10.1016/j.redox.2020.101638 32863203 PMC7381685

[B8] Jiménez-Jiménez C. Moreno-Borrallo A. Dumontel B. Manzano M. Vallet-Regí M. (2023). Biomimetic camouflaged nanoparticles with selective cellular internalization and migration competences. Acta Biomater. 157, 395–407. 10.1016/j.actbio.2022.11.059 36476646

[B9] Kırımlıoğlu G. Y. (2023). History, Introduction, and Properties of PLGA as a Drug Delivery carrier Poly (lactic-co-glycolic Acid)(Plga) Nanoparticles for Drug Delivery. Elsevier, 3–25.

[B10] La Via L. Sangiorgio G. Stefani S. Marino A. Nunnari G. Cocuzza S. (2024). The global burden of sepsis and septic shock. Epidemiologia 5 (3), 456–478. 10.3390/epidemiologia5030032 39189251 PMC11348270

[B11] Li W. Li D. Chen Y. Abudou H. Wang H. Cai J. (2022). Classic signaling pathways in alveolar injury and repair involved in sepsis‐induced ALI/ARDS: new research progress and prospect. Dis. Markers 2022 (1), 6362344–6362349. 10.1155/2022/6362344 35726235 PMC9206211

[B12] Li Y. Ai S. Li Y. Ye W. Li R. Xu X. (2025). The role of natural products targeting macrophage polarization in sepsis-induced lung injury. Chin. Med. 20 (1), 19. 10.1186/s13020-025-01067-4 39910395 PMC11800549

[B13] Liu Z. Ting Y. Li M. Tan Y. Long Y. (2024). From immune dysregulation to organ dysfunction: understanding the enigma of Sepsis. Front. Microbiol. 15, 1415274. 10.3389/fmicb.2024.1415274 39252831 PMC11381394

[B14] Madamsetty V. S. Mohammadinejad R. Uzieliene I. Nabavi N. Dehshahri A. García-Couce J. (2022). Dexamethasone: insights into pharmacological aspects, therapeutic mechanisms, and delivery systems. ACS Biomaterials Science and Engineering 8 (5), 1763–1790. 10.1021/acsbiomaterials.2c00026 35439408

[B15] Puricelli C. Gigliotti C. L. Stoppa I. Sacchetti S. Pantham D. Scomparin A. (2023). Use of poly lactic-co-glycolic acid nano and micro particles in the delivery of drugs modulating different phases of inflammation. Pharmaceutics 15 (6), 1772. 10.3390/pharmaceutics15061772 37376219 PMC10301392

[B16] Qiao X. Yin J. Zheng Z. Li L. Feng X. (2024). Endothelial cell dynamics in sepsis-induced acute lung injury and acute respiratory distress syndrome: pathogenesis and therapeutic implications. Cell Commun. Signal. 22 (1), 241. 10.1186/s12964-024-01620-y 38664775 PMC11046830

[B17] Rodríguez-Morales P. Franklin R. A. (2023). Macrophage phenotypes and functions: resolving inflammation and restoring homeostasis. Trends Immunol. 44 (12), 986–998. 10.1016/j.it.2023.10.004 37940394 PMC10841626

[B18] Savchenko I. V. Zlotnikov I. D. Kudryashova E. V. (2023). Biomimetic systems involving macrophages and their potential for targeted drug delivery. Biomimetics 8 (7), 543. 10.3390/biomimetics8070543 37999184 PMC10669405

[B19] Sun B. Lei M. Zhang J. Kang H. Liu H. Zhou F. (2023). Acute lung injury caused by sepsis: how does it happen? Front. Medicine 10, 1289194. 10.3389/fmed.2023.1289194 38076268 PMC10702758

[B20] Tang S. Mao X. Chen Y. Yan L. L. Ye L. P. Li S. W. (2022). Reactive oxygen species induce fatty liver and ischemia-reperfusion injury by promoting inflammation and cell death. Front. Immunology 13, 870239. 10.3389/fimmu.2022.870239 35572532 PMC9098816

[B21] Wang Z. Wang Z. (2023). The role of macrophages polarization in sepsis-induced acute lung injury. Front. Immunology 14, 1209438. 10.3389/fimmu.2023.1209438 37691951 PMC10483837

[B22] Wang W.‐T. Zhang Y.‐Y. Li Z.‐R. Li J.‐M. Deng H.‐S. Li Y.‐Y. (2024). Syringic acid attenuates acute lung injury by modulating macrophage polarization in LPS-Induced mice. Phytomedicine 129, 155591. 10.1016/j.phymed.2024.155591 38692075

[B23] Wang L. Wang K. Zheng H. (2025). Macrophage membrane-coated nanocarriers in myocardial infarction: a paradigm shift in targeted cardiac therapy. Int. J. Nanomedicine 20, 13499–13525. 10.2147/IJN.S546817 41229674 PMC12604587

[B24] Woodworth K. E. Callaghan N. I. Davenport Huyer L. (2026). Biomaterial strategies for targeted intracellular delivery to phagocytes. Adv. Funct. Mater. 36 (1), e08761. 10.1002/adfm.202508761

[B25] Wu Y. Wan S. Yang S. Hu H. Zhang C. Lai J. (2022). Macrophage cell membrane-based nanoparticles: a new promising biomimetic platform for targeted delivery and treatment. J. Nanobiotechnology 20 (1), 542. 10.1186/s12951-022-01746-6 36575429 PMC9794113

[B26] Zhang Y. Long Y. Wan J. Liu S. Shi A. Dan L. (2023). Macrophage membrane biomimetic drug delivery system: for inflammation targeted therapy. J. Drug Target. 31 (3), 229–242. 10.1080/1061186X.2022.2071426 35587560

[B27] Zhang J. Yan W. Dong Y. Luo X. Miao H. Maimaijuma T. (2024a). Early identification and diagnosis, pathophysiology, and treatment of sepsis-related acute lung injury: a narrative review. J. Thorac. Dis. 16 (8), 5457–5476. 10.21037/jtd-24-1191 39268131 PMC11388254

[B28] Zhang Z. Li M. Zhang X. Zhou F. (2024b). Novel strategies for tumor treatment: harnessing ROS-Inducing active ingredients from traditional chinese medicine through multifunctional nanoformulations. Int. Journal Nanomedicine 19, 9659–9688. 10.2147/IJN.S479212 39309188 PMC11416109

[B29] Zhang Z. Ma T. Liu Q. Nan J. Liu G. Yang Y. (2025). Exosomes derived from bone marrow mesenchymal stem cells encapsulated in M2 macrophage cell membrane targeted to inhibit joint periprosthetic inflammation. ACS Appl. Mater. and Interfaces 17 (15), 22279–22292. 10.1021/acsami.4c22304 40168527

[B30] ZhengS. WangZ. XueY. ZhangH. XiaoY. HuY. (2026). The postnatal lung maturation disrupted by increased pulmonary blood flow and its clinical implications. JACC Asia. 10.1016/j.jacasi.2026.02.013 41885686

