# Supplementary material for: Biomimetic dexamethasone-loaded nanoparticles attenuate sepsis-induced acute lung injury
Source: Front Pharmacol. 2026 Jul 29;17:1857409. doi: 10.3389/fphar.2026.1857409 (PMC13461071; doi:10.3389/fphar.2026.1857409)
Supplement: Supplementary file 1 [file DataSheet1.pdf]

Biomimetic Dexamethasone-Loaded Nanoparticles Attenuate Sepsis-Induced Acute Lung Injury

LiuGuang Song[abc],ZheChu Xuan[d],ChunNa Jin[abc], LiangLiang Jia[abc], Nan Zheng[d], YuMeng Liu[g], ZhengMing Wu[e*],Jian Shen[abc*] , Huijun Liang[f*]

a, Department of Cardiology, The Second Affiliated Hospital, Zhejiang University School of Medicine, Hangzhou, 310000, China

b, State Key Laboratory of Transvascular Implantation Devices, Hangzhou, 310000, China

c, Heart Regeneration and Repair Key Laboratory of Zhejiang Province, Hangzhou, 310009, China

d,School of Medicine & Nursing, Huzhou University, Huzhou, 313000, China

e, The Third People's Hospital of Deqing, Deqing Hospital of Hangzhou Normal University, Deqing, 313200, China

f, Laboratory Department, The Second Hospital of Jiaxing, JiaXing, 314001, China

g, Zhejiang Provincial People's Hospital, Affiliated People's Hospital, Hangzhou Medical College, Hangzhou, 310014, China

[*]Correspondence should be addressed to wzm006@163.com, shenjian27753@zju.edu.cn and jxfbylhj@163.com

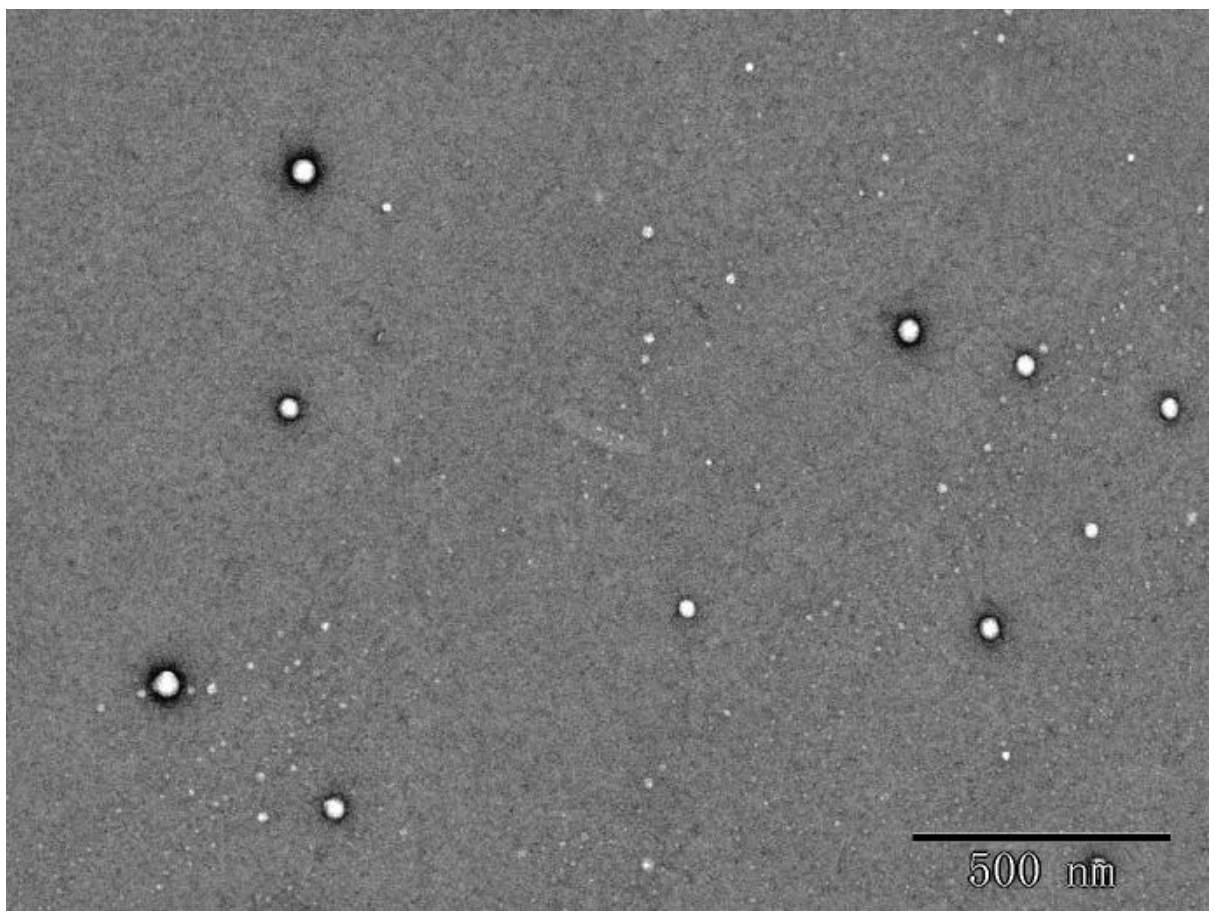

Figure S1 TEM image of Dex@PLGA

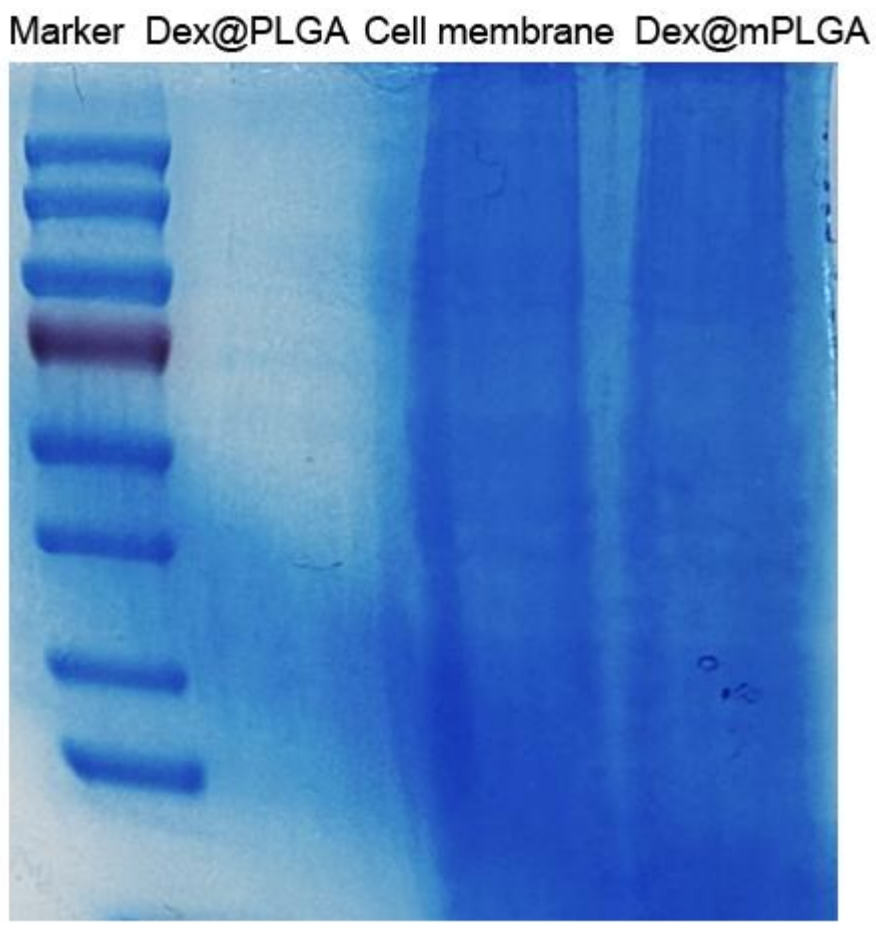

Figure S2 SDS-PAGE analysis of RAW264.7 membrane protein retention in Dex@mPLGA. Lanes show the protein marker, uncoated Dex@PLGA, cell membrane lysate, and cell membrane-coated Dex@mPLGA.

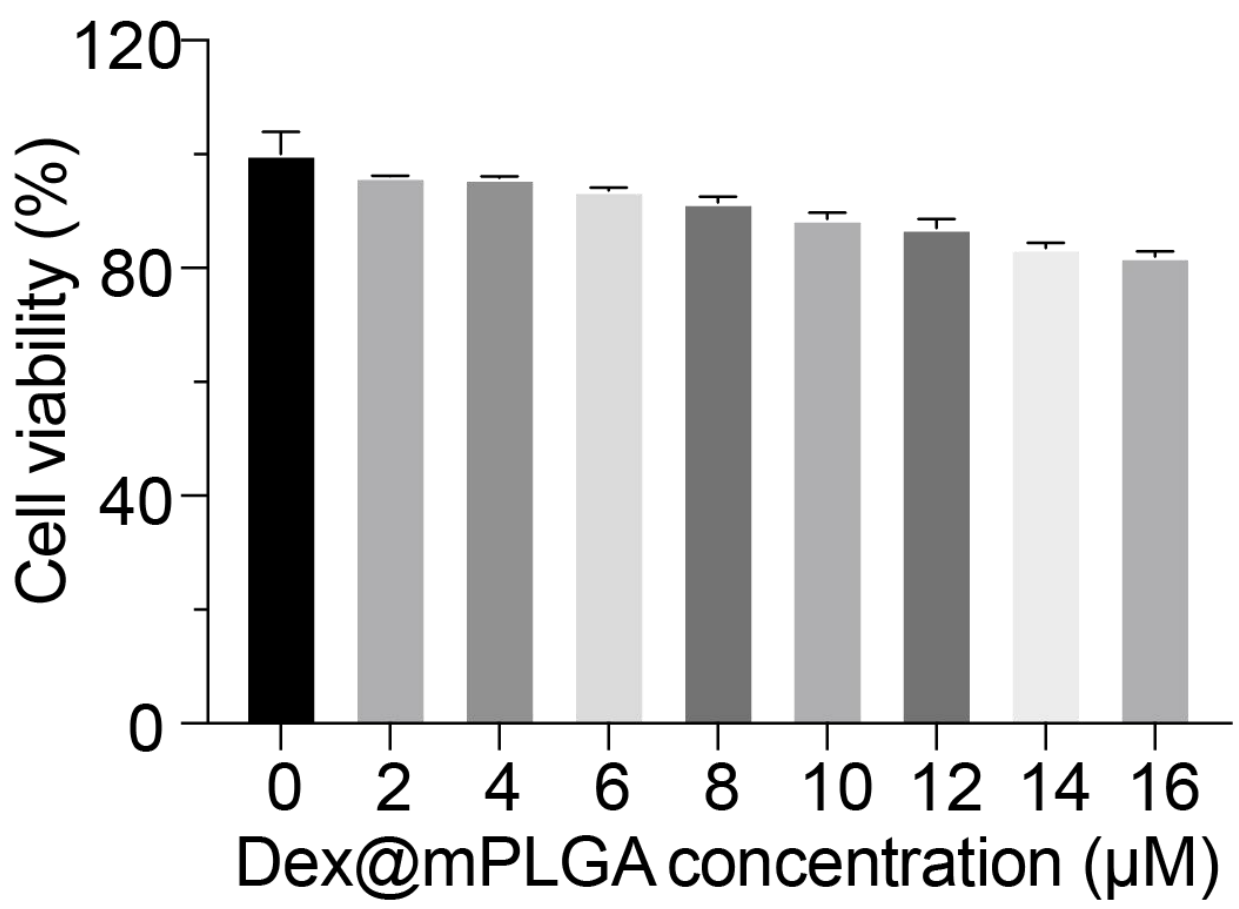

Figure S3 Cytotoxicity evaluation of Dex@mPLGA in RAW264.7 cells. Cells were treated with various concentrations (0–16 μM) of Dex@mPLGA for 24 h, and cell viability was assessed by CCK-8 assay. Data are presented as mean ± SD (n = 3). The results indicate that cell viability remained above 80% even at the highest concentration, confirming the low intrinsic cytotoxicity

of the nanocarrier.

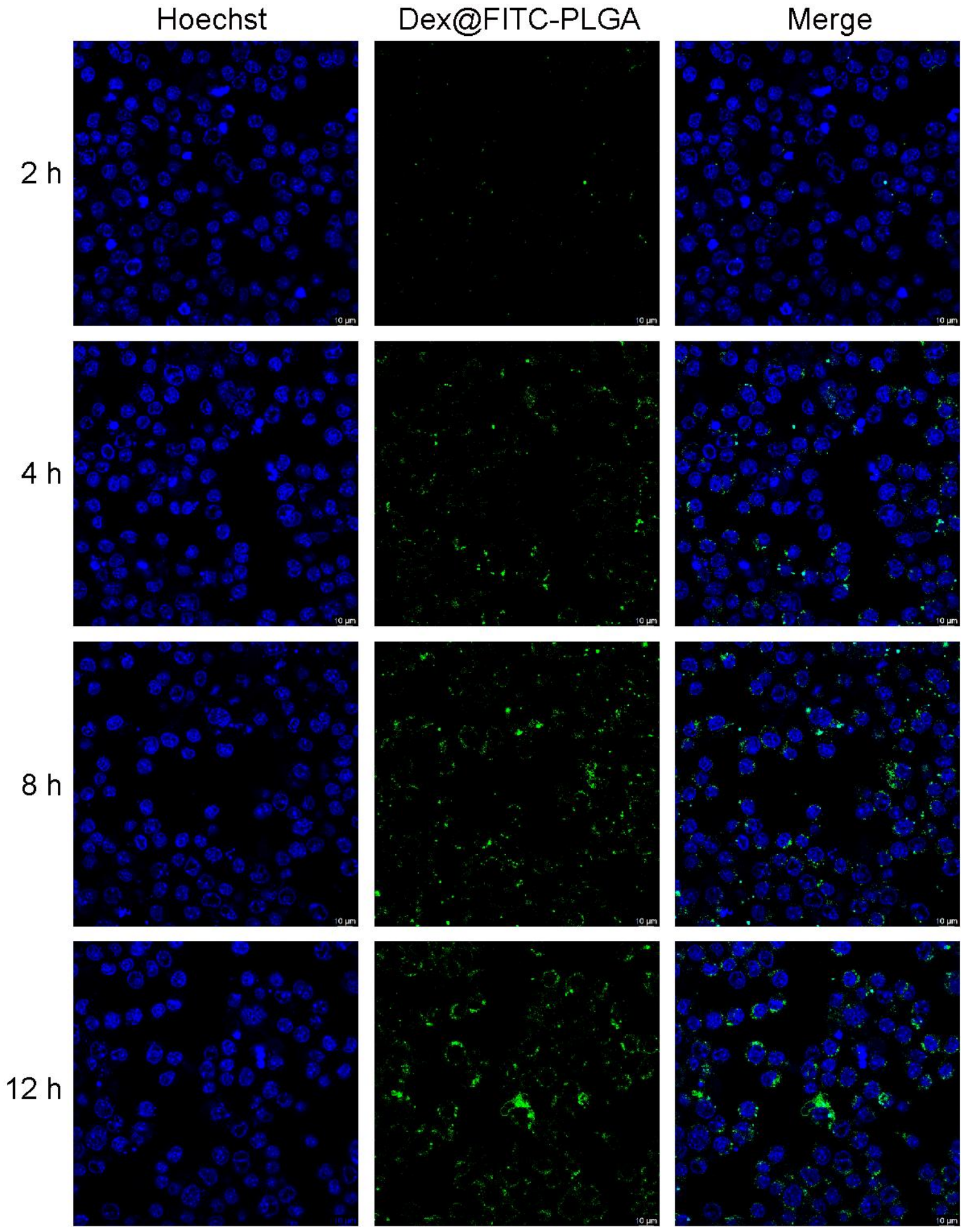

Figure S4 Cellular uptake of FITC-labeled Dex@PLGA by RAW264.7 (n=3).

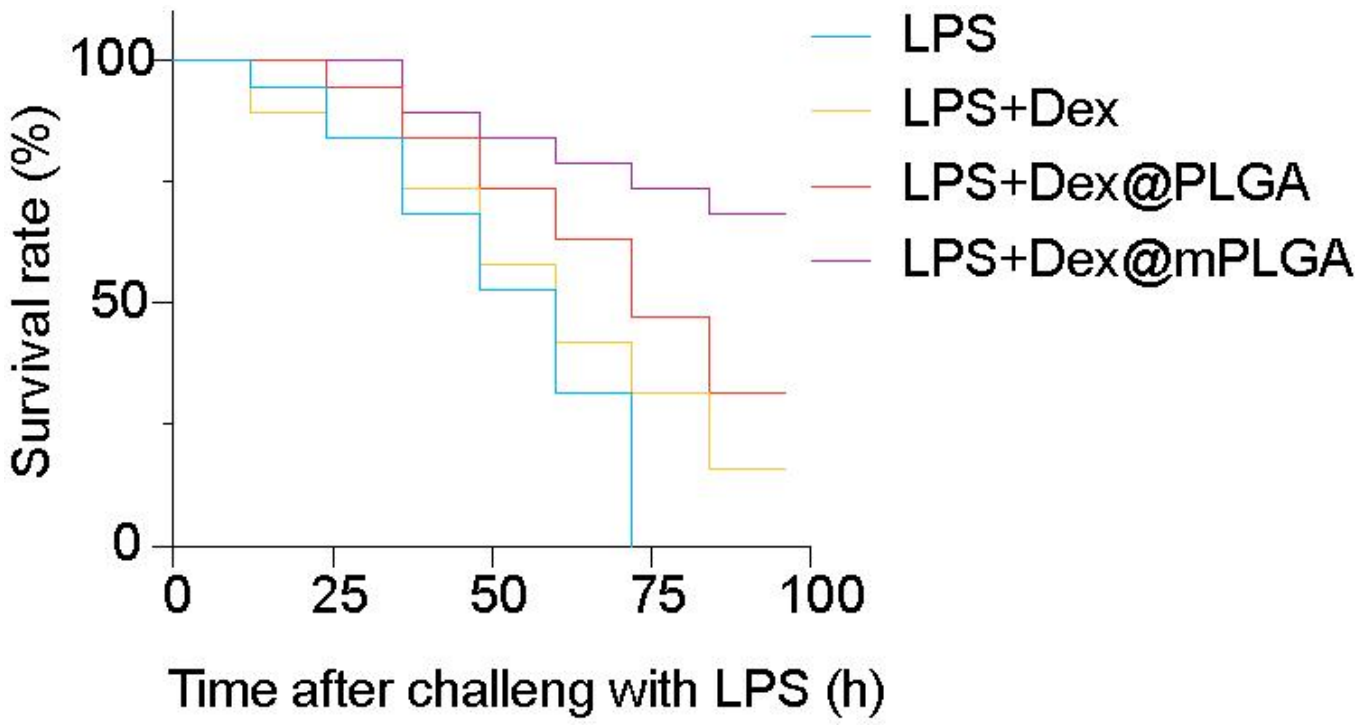

Figure S5 Survival curves of mice in different treatment groups (n=10).

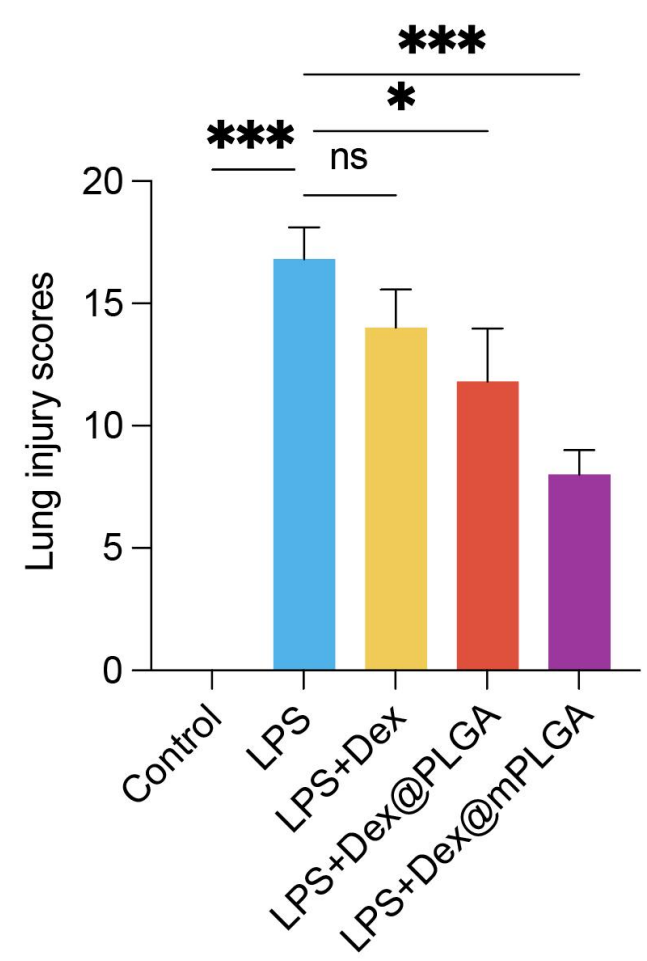

Figure S6 Semiquantitative histological lung-injury scores based on H&E-stained lung tissue sections from the indicated experimental groups. Lung injury was scored according to inflammatory-cell infiltration, hemorrhage, and edema (n=5). Data are presented as mean ± SD. *p < 0.05, **p < 0.01, ***p < 0.001.

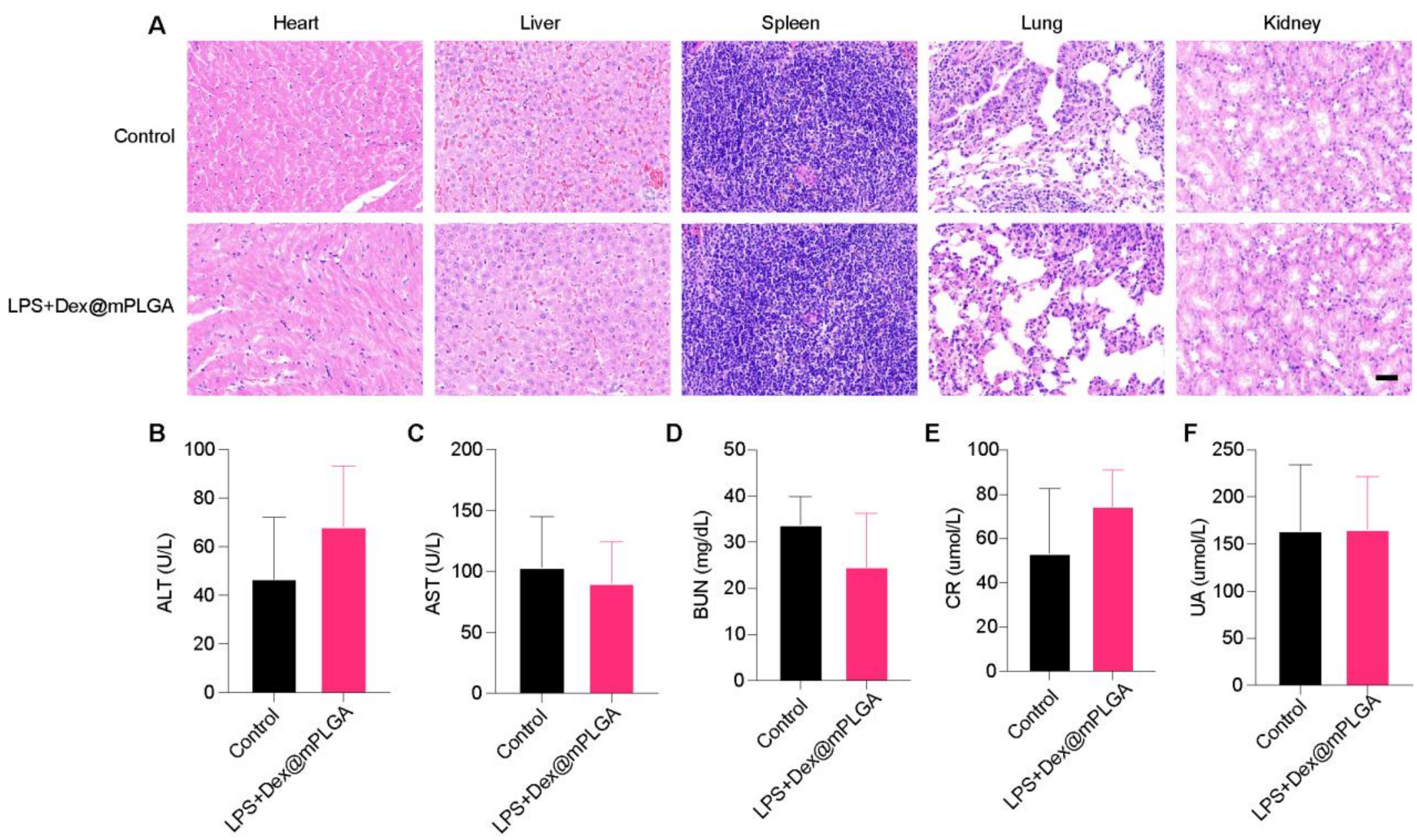

Figure S7. In vivo biosafety evaluation of Dex@mPLGA after 7 days of administration. (A) H&E staining of major organs (heart, liver, spleen, lung, kidney) from control and Dex@mPLGA-treated mice. Scale bar = 100 μm. (B–F) Serum biochemical parameters: alanine aminotransferase (ALT, B), aspartate aminotransferase (AST, C), blood urea nitrogen (BUN, D), creatinine (Cr, E), and uric acid (UA, F). Data are presented as mean ± SD (n = 5). No significant differences were observed between the Dex@mPLGA group and the control group.
